# Philadelphia Association for Medical Instruction

**Published:** 1849

**Authors:** 


### ARTICLE VIII.

### PHILADELPHIA ASSOCIATION FOR MEDICAL INSTRUCTION.

The seventh annual announcement of this Institution is on our table. We are highly pleased with the plan of Instruction proposed and hope our friends may have a full class. The lectures commence on. the first Monday in April next, and continue until the first of October, when the regular winter courses in the colleges commence, with a mid-summer's recess. Three lectures will be given daily, and the course embraces instruction in the several departments of the science of Medicine, as follows:

*Anatomy*—J. M. Allen, M. D.
*Physiology*—F. J. Smith, M. D.
*Materia Medica and Therapeutics*—Francis West, M. D.
*Medical Chemistry*---Robert Bridges, M. D.
*Diseases of Children*—J. Forsyth Meigs, M. D.
*Institutes and Practice of Surgery*---J. M. Wallace, M. D.
*Pathology and Practice of Medicine*---Alfred Stillé, M. D.
*Obstetrics and Diseases of Women*--David H. Tucker, M. D.

Fee for Course, \$60 ; single tickets, \$10. E.

